# Purinergic Signaling in the Regulation of Gout Flare and Resolution

**DOI:** 10.3389/fimmu.2021.785425

**Published:** 2021-12-01

**Authors:** Xiaoling Li, Jie Gao, Jinhui Tao

**Affiliations:** Department of Rheumatology and Immunology, The First Affiliated Hospital of University of Science and Technology of China (USTC), Division of Life Sciences and Medicine, University of Science and Technology of China, Hefei, China

**Keywords:** purinergic signaling, gout flare, ATP, Adenosine, P2X_7_R, IL-1β

## Abstract

Gout flares require monosodium urate (MSU) to activate the NLRP3 inflammasome and secrete sufficient IL-1β. However, MSU alone is not sufficient to cause a flare. This is supported by the evidence that most patients with hyperuricemia do not develop gout throughout their lives. Recent studies have shown that, besides MSU, various purine metabolites, including adenosine triphosphate, adenosine diphosphate, and adenosine bind to different purine receptors for regulating IL-1β secretion implicated in the pathogenesis of gout flares. Purine metabolites such as adenosine triphosphate mainly activate the NLRP3 inflammasome through P2X ion channel receptors, which stimulates IL-1β secretion and induces gout flares, while some purine metabolites such as adenosine diphosphate and adenosine mainly act on the G protein-coupled receptors exerting pro-inflammatory or anti-inflammatory effects to regulate the onset and resolution of a gout flare. Given that the purine signaling pathway exerts different regulatory effects on inflammation and that, during the inflammatory process of a gout flare, an altered expression of purine metabolites and their receptors was observed in response to the changes in the internal environment. Thus, the purine signaling pathway is involved in regulating gout flare and resolution. This study was conducted to review and elucidate the role of various purine metabolites and purinergic receptors during the process.

## Introduction

Gout is an inflammatory disease that manifests clinically as redness, swelling, and pain in the joints. Research involving multiple ethnicities has reported that the prevalence of gout in adults ranges between 0.68% and 3.90% (1). With the development of the economy, the incidence of gout is increasing every year, while the social danger of the disease is becoming a growing concern among scholars. Hyperuricemia underlies the pathogenesis of gout, with supersaturated uric acid being deposited in the joint cavity to form monosodium urate (MSU). The MSU stimulates abnormal IL-1β secretion to cause gout flares, which is achieved through recognition by Toll-like receptors (TLRs) or NOD-like receptors (NLRs) to activate the innate immune system (1). The activation of NLRP3 inflammasome releases large amounts of IL-1β is a central process of MSU-mediated gout flares (2). However, clinical studies have shown that most patients with hyperuricemia do not experience gout flares (3). Additionally, the presence of triggering factors, including alcohol abuse, overeating, and exertion, is often required for a gout flare, which suggests that MSU alone is not sufficient to induce gout flares, and there is a need for other causative signals to act *in vivo* for gout flares.

The activation of NLRP3 inflammasome and subsequent induction of the release of IL-1β exerts a central role in the initiation of gout flares. Besides MSU, extracellular DAMPs and PAMPs, including bacteria, viruses, and adenosine triphosphate (ATP), are known to be responsible for the activation of the NLRP3 inflammasome. Among them, ATP-mediated activation of the P2X_7_R-NLRP3 signaling pathway has gradually been recognized in the pathogenesis of gout (4, 5). Following tissue necrosis caused by MSU deposition, local ATP arising from the cellular release is increased, which can activate the P2X_7_ receptor leading to intra-and extracellular ion flow, activation of the NLRP3 inflammasome, and secretion of IL-1β, ultimately triggering a gout flare. Furthermore, gene polymorphisms in the P2X_7_ receptor have been shown to regulate IL-1β secretion *in vivo*. Several studies have demonstrated that a gain-in-function of the P2X_7_ receptor is associated with an elevated rate of gout flares (6–8). Moreover, colchicine, a classical drug for treating acute gout flares, was found to inhibit the activation of ATP-induced P2X_7_ receptor (9), which could be the mechanism of action against gout. This indirectly confirmed the role of ATP and P2X_7_ receptor in gout resolution. Therefore, as an important messenger of the purinergic pathway, ATP is considered a second pathogenic signal for gout.

Besides ATP, other purinergic metabolites, such as adenosine diphosphate (ADP) and adenosine, can bind to the corresponding purinergic receptors affecting IL-1β secretion in gout. However, the purine signaling pathway exerts different regulatory effects on inflammation. For example, in contrast to ATP and ADP, adenosine exerts an inhibitory effect on inflammation *in vivo* (10). In addition, the expression of purine metabolites and their receptors varies in response to changes in the inflammatory internal environment during a gout flare. MSU was found to regulate the expression of the P2X_7_ receptor (11). Thus, the interaction between purinergic metabolites and purinergic receptors mediates the intracellular signaling communication, which jointly regulates the onset and resolution of gout flares. In this article, we have reviewed the possible regulatory role of the purinergic signaling pathway in gout flares to understand and combat gout in a more precise manner.

## Classification and Function of Purinergic Receptors

The existence of purinergic receptors in the body was first proposed by Burnstock (12) in 1972. Since then, an increasing number of purinergic receptors have been identified, and researchers have gradually noticed the role of purinergic signaling pathways in the biological effects of diseases. The purinergic receptors, also known as P receptors, are classified as P1 and P2 receptors based on their ligands; the former binds to adenosine while the latter binds to ATP, ADP, uridine triphosphate (UTP), and uridine diphosphate (UDP). The P1 receptors are G protein-coupled receptors, which can be classified into four subtypes (A_1_, A_2A_, A_2B_, and A_3_) depending on the structure of the G proteins and intracellular conductance. The A_2A_ and A_2B_ receptors preferentially couple to G_s_ and exert their effects *via* the AC-cAMP-PKA pathway while the A_1_ and A_3_ subtypes couple to G_i_ and inhibit the AC activity (13). The P2 receptors, on the other hand, are divided into two major groups, P2X and P2Y receptors, depending on the signal transduction pathway. The P2X receptors are ligand-gated ion channel receptors that recognize ATP. Upon activation, the pore channels open and allow the flow of extracellular Na^+^, Ca^2+^, K^+^, and the entry of macromolecules. Seven P2X receptors have been identified so far (P2X_1–7_ receptors). The P2Y receptors are G protein-coupled receptors that primarily recognize ATP, UTP, and its metabolites. They can be further classified into P2Y_1_-like receptors (coupled to G_q_, including P2Y_1_, P2Y_2_, P2Y_4_, P2Y_6_, and P2Y_11_ receptors) and P2Y_12_-like receptors (coupled to G_i_, including P2Y_12_, P2Y_13_, and P2Y_14_ receptors) based on the intracellular signaling proteins coupled to the G proteins (14). The former receptors exert their effect *via* the PLC-IP3/DAG-PKC pathway while the latter inhibits the activity of AC, thus, producing different biological effects (**Supplementary Table 1**).

The purinergic receptors are distributed in almost all cell types, and upon recognition of the corresponding ligands, they mediate a variety of biological effects and enable the development of many diseases, including cardiovascular (15, 16), metabolic (17, 18), neurological diseases (19), and cancer (20, 21). In inflammatory diseases, purinergic receptors play an important regulatory role and participate in immune- (22), infectious- (23), and neurotransmission (24)-mediated inflammatory responses to maintain homeostasis in the organism. In the regulation of inflammation, the role of different purine signals varies. For example, the activation of P2X_7_ receptors promotes the inflammatory response, whereas the activation of P1 receptors inhibits it. Therefore, the P receptors can coordinate the initiation, persistence, and resolution of the inflammatory response (25). Gout is an inflammatory disease caused by abnormal purine metabolism. The purine metabolites exert complex regulatory effects on the inflammatory response, influencing the onset and resolution of gout to varying degrees.

## Effect of Purinergic Signaling Pathway on Gout Flares

Gout flares are a result of a complex acute inflammatory response, where the macrophages recognize MSU deposited on the joint surface, leading to the secretion of IL-1β. This is thought to be the central process in the initiation of gout flares (26). Immediately after the release of IL-1β, the inflammatory cascade response induces neutrophil accumulation in the joints, thus, exacerbating gout flare (27). Therefore, overproduction of IL-1β is a central link in the MSU-induced gouty joint inflammation. It is essential to explore the mechanism of activation of IL-1β for investigating the pathogenesis of gout flares, and it is found that the purinergic signaling pathway is involved in the regulation of IL-1β secretion in gout.

### Activation of the P2X Receptor Signaling Pathway Leads to the Promotion of Gout Flares

The P2X receptors are ATP-gated ion channel receptors, and of these P2X receptors, the P2X_7_ receptor, initially defined as the P2Z receptor, possesses a unique sequence and rich function despite being the latest identified (28). Activation of the P2X_7_ receptor by ATP opens ion channels causing Ca^2+^ influx and K^+^ outflux to activate the NLRP3 inflammasome in the innate immune system, where Ca^2+^ may represent the second messenger of inflammasome activation (29). Also, the P2X_7_ receptor has a unique structure and function, which does not only form ion channels but also forms the pore channels, allowing the passage of up to 900 Da molecules upon continuous activation by high concentrations of ATP. The formation of such pores appears to be required to activate the NLRP3 inflammasome (30, 31). Upon activation, the NLRP3 proteins interact with apoptosis-associated speck-like protein containing a CARD (ASC), which then recruits the pro-caspase-1 forming the NLRP3 inflammasome, which subsequently undergoes cleavage to form active caspase-1. This is further processed, leading to the secretion of mature IL-1β and IL-18 that exert inflammatory effects (32). Following pharmacological inhibition of the P2X_7_ receptor, the level of ATP-induced IL-1β release is reduced in inflammatory cells (33). A similar reduction was observed in the levels of the P2X_7_ receptor, ASC, or NLRP3-deficient mouse macrophages. These findings suggested that the activation of the ATP-mediated P2X_7_ receptor plays a crucial role in promoting IL-1β secretion in inflammatory cells (34). Currently, it is recognized that the P2X_7_ receptor, among the family of P2XRs, is the most relevant in the inflammatory response.

Gout is an acute inflammatory disease based on hyperuricemia. Predisposing factors, including alcohol abuse, overeating, cold, and late nights, are required to initiate a gout flare. We evaluated the pathogenic signals embedded in these factors associated with gout flares and found that under these conditions, ATP fluctuations were observed *in vivo*. This, for example, included mechanical stimulation during strenuous exercise promotes ATP release (35), and the adaptive febrile response of the body during cold increases ATP (36). Dramatic changes in ATP levels can activate the P2X_7_R-NLRP3 signaling pathway and promote the IL-1β secretion associated with gout flares. Thus, ATP is considered to be involved in gout flares. A study by Tao et al. observed no difference in the IL-1β concentrations in peripheral blood leukocyte cultures from gout and hyperuricemia patients when stimulated with MSU alone, but upon stimulation with MSU and ATP together, IL-1β concentrations were found to be significantly higher in gout patients than that of the hyperuricemia patients (6). Thus, it is suggested that ATP is the second pathogenic signal for gout besides MSU. Importantly, the ability of the P2X_7_ receptor to promote IL-1β secretion upon activation may be a major determinant of gout flares, which is influenced by the gene polymorphisms of the P2X_7_ receptor. Studies have confirmed that gout patients possess single nucleotide polymorphism in functionally enhanced P2X_7_ receptor compared to hyperuricemia patients (37), thus establishing an essential role of P2X_7_ receptor in gout flares.

In hyperuricemic patients, synergistic action between MSU and ATP is required to fully activate the NLRP3 inflammasome, which causes sufficient secretion of IL-1β, leading to the onset of gout flares. Thus, it can be argued that as a receptor for ATP, the P2X_7_ receptor is an important regulator in determining gout flares, explaining why most hyperuricemic patients do not develop gouty arthritis throughout their lives.

Besides the P2X_7_ receptor, the P2X_4_ receptor can also be activated by ATP, causing an inflammatory response, which is functionally similar to the P2X_7_ receptor. Upon activation, it forms large-conductance pores in the cell membrane facilitating the ion flow, which subsequently activates the NLRP3 inflammasome. This further enhances the body’s inflammatory response through its synergy with the P2X_7_ receptor (38).

### Different Roles of Activated P2Y Receptor Signaling Pathway in Gout Flares

P2Y receptors belong to the G protein-coupled receptor family and are further divided into P2Y_1_-like receptors (coupled to G_q_) and P2Y_12_-like receptors (coupled to G_i_), which exert regulatory effects on inflammation by affecting the activity of PLC and AC, respectively (39). Upon recognizing the corresponding nucleotide, P2Y_1_-like receptors coupled to G_q_ activate the PLC-IP3/DAG-PKC pathway, promoting Ca^2+^ mobilization and inflammatory response. Also, the P2Y_1_-like receptors can activate AC; for example, the P2Y_11_ receptor promotes the activation of the AC-cAMP-PKA pathway to initiate the inflammatory response (40). The P2Y_12_-like receptors coupled to G_i_ can exert an inhibitory effect on AC, further reducing the conversion of dephosphorylation of ATP to cAMP, thus, inhibiting the cAMP-PKA pathway-mediated inflammatory response.

The P2Y_1_-like receptors, including the P2Y_1_, P2Y_2_, P2Y_4_, P2Y_6_, and P2Y_11_ receptors, can promote inflammation either through the PLC-IP3/DAG-PKC pathway or through the activation of AC. Among these P2Y_1_-like receptors, the P2Y_2_ receptor may be closely associated with gout flares. Besides its inflammatory function, the P2Y_2_ receptor can activate the NLRP3 inflammasome by stimulating the action of ATP release to further activate the P2X_7_ receptor (41).

The P2Y_2_ receptors are expressed on various cells, including macrophages, lymphocytes, neutrophils, and eosinophils, and are activated by multiple nucleotides such as ATP and UTP to participate in immune regulation and inflammatory responses. The activated P2Y_2_ receptors exhibit upregulated expression of inflammatory cytokines and chemokines, while the release of inflammatory factors is blocked by the treatment used to inhibit the P2Y_2_ receptors (42, 43). Furthermore, the activated P2Y_2_ receptors open pannexin-1 channels by inducing PLC activation and Ca^2+^ mobilization, which leads to further release of ATP promoting IL-1β maturation and secretion through the P2X_7_R-NLRP3 pathway (41). Thus, the binding of the P2Y_2_ receptors to ligands promoted the release of inflammatory factors and initiated the inflammatory response in multiple ways. The absence of the P2Y_2_ receptor is observed to reverse the increase of the nucleotide-induced IL-1β release (44).

The current study suggested that the P2Y_6_ receptor activated by UDP and UTP is involved in the pathogenesis of gout. Uratsuji et al. found that the P2Y_6_ receptors were involved in the MSU-induced inflammatory responses. The activation of the P2Y_6_ receptors in a variety of cells was observed to contribute to the production of inflammatory cytokines; particularly, the inhibition of the P2Y_6_ receptor in THP-1 cells could reduce MSU-induced IL-1β production (45). Additionally, the P2Y_6_ receptor affected the MSU-induced neutrophil function associated closely with gout. Sil et al. found that the inhibition of the P2Y_6_ receptors reduced neutrophil migration and IL-8 production, which is responsible for recruiting neutrophils to the joint cavity during a gout flare, thereby amplifying the effects of gout (46). In addition, treatment with the P2Y_6_ receptor antagonist MRS2578 inhibited the formation of MSU-induced neutrophil extracellular traps (NETs) in gout (46). Therefore, activation of the P2Y_6_ receptor may contribute to the persistence of gout flares.

The P2Y_12_-like receptors coupled to G_i_, including P2Y_12_, P2Y_13,_ and P2Y_14_ receptors, inhibit the cAMP-PKA pathway-mediated inflammatory response and theoretically inhibit gout pathogenesis. However, due to the complexity of the mechanisms of action in different internal environments, the regulation of inflammation by P2Y_12_-like receptors is also diverse. For example, cAMP has long been considered an inducer of inflammation. Activation of the cAMP-PKA-ERK signaling pathway promotes IL-1β secretion (47). Additionally, the AC/cAMP/PKA upregulates IL-1β-induced IL-6 production by enhancing the JAK2/STAT3 pathway (48). However, cAMP has also been recently pointed out as an important factor in regulating inflammation relief (49). The cAMP inhibits NF-κB transcription by activating downstream PKA to reduce the production of pro-inflammatory factors. Also, it promotes the phosphorylation of CENP to increase the production of anti-inflammatory factors and stimulate macrophage polarization. Moreover, cAMP has been found to inhibit the assembly of the NLRP3 inflammasome by directly binding to NLRP3 proteins, and promotes their ubiquitination and degradation (50, 51), thus acting as a negative regulator of the NLRP3 inflammasome reducing the secretion of IL-1β. Therefore, the P2Y_12_-like receptors can not only exert inhibitory inflammatory effects but can also often exhibit pro-inflammatory effects through the regulation of cAMP. In microglia, extracellular ADP was observed to act on P2Y_12_ receptors that activated NF-κB and NLRP3 inflammasomes while the inhibition of the P2Y_12_ receptors reduced the IL-1β levels (52). Similarly, the P2Y_14_ receptors promoted the secretion of inflammatory factors IL-1α, IL-8, and IL-6 in response to MSU stimulation (53).

Complex regulatory effects of inflammation are manifested in the P2Y_14_ receptor. In a gout study, Li et al. found that the P2Y_14_ receptors on macrophages negatively regulated cAMP, which enhanced the MSU-induced activation of the NLRP3 signaling pathway. The knockdown of the P2Y_14_ receptor then limited the activation of the NLRP3 inflammasome, which in turn attenuated the MSU-induced inflammatory infiltration of synovial tissue (54). Thus, in acute gouty arthritis, the activation of the P2Y_14_ receptor can exert a pro-inflammatory effect through the cAMP/NLRP3 signaling pathway.

The P2Y receptors in gout often play conflicting roles both in promoting remission of inflammation and maintaining the persistence of inflammation. As mentioned earlier, the P2Y_12_-like receptor signaling pathway that inhibits inflammation can have pro-inflammatory effects through activation of NLRP3 inflammasome. Similarly, the P2Y_1_-like receptor signaling pathway that promotes inflammation may exhibit an inhibitory effect on inflammation. For example, in a gout mouse model, the activation of the P2Y_6_ receptors reduces 1-palmitoyl-2-linoleyl-3-acetyl-rac-glycerol-induced neutrophil infiltration, alleviating joint symptoms (55). Thus, the P2Y receptors play a complex role in inflammatory diseases, either as a friend or foe. Besides modulating the pro-inflammatory factors in the regulation of inflammatory responses, P2Y receptors can also influence the expression of anti-inflammatory cytokines. TGF-β and IL-10 are the major cytokines responsible for gout resolution (56). The P2Y_1_ receptors were found to enhance the effects of TGF-β1 and IL-10 (57, 58), while the P2Y_11_ receptor, although reported to inhibit TGF-β1 production through activation of cAMP, was found to upregulate the IL-10 expression (57, 58). Overall, the data suggested that the P2Y receptor signaling pathway was involved in the pathogenesis of gout in different ways.

### Activation of the P1 Receptor Signaling Pathway Inhibits Inflammation in Gout Flares

The P1 receptors belong to the family of G protein-coupled receptors and include A_1_, A_2A_, A_2B,_ and A_3_ receptors. These are widely expressed on immune cells and exhibit anti-inflammatory effects. The ligand of the P1 receptor, adenosine, is the main product of sequential hydrolysis of extracellular ATP, which is catalyzed by CD39 and CD73. Under physiological conditions, the adenosine concentration is maintained at low levels, but under certain conditions such as inflammation, hypoxia, and cellular injury, the concentration of adenosine is increased by degrading extracellular ATP and ADP. Adenosine is then rapidly metabolized to AMP and inosine by the action of adenosine kinase or adenosine deaminase. The contribution of adenosine in alleviating inflammation and maintaining homeostasis in the organism has been recognized widely (10).

The P1 receptor signaling exerts anti-inflammatory effects through a variety of pathways. The adenosine-activated P1 receptors (A_1_, A_3_) increase the inhibition of neutrophil adhesion, reducing the production of superoxide and secretion of inflammatory cytokines (59–61). Furthermore, the interaction of adenosine with A_1_, A_2A_, and A_3_ receptors inhibits the release of IL-1β from activated human peripheral blood mononuclear lymphocytes (62). Also, the interaction of adenosine with A_3_ receptors was found to reduce TNF-α production in the macrophage cell lines (63), while the interaction of adenosine with A_1_ receptors was found to reduce parasite-stimulated production of reactive oxygen species (ROS) and IL-8 from neutrophils (64). Besides its inhibitory effect on the secretion of inflammatory factors, the activation of the P1 receptor signaling pathway also promotes the secretion of anti-inflammatory cytokines, including TGF-β1 and IL-10, by the immune cells (57, 60).

The targeting of adenosine receptors has been efficient in the treatment of several inflammatory diseases. The activation of adenosine-mediated A_2A_ receptor contributed to block the activity of NLRP3 inflammasome, thereby reducing the production of pro-inflammatory cytokines while increasing the production of anti-inflammatory cytokines (65, 66). In post-traumatic brain injury events, agonizing the A_3_ adenosine receptor could improve the neurocognitive function by inhibiting the activation of the NLRP3 inflammasome (67). Similarly, in arthritic mice, activating the A_3_ adenosine receptors could reduce the production of TNF-α and alleviate arthritic symptoms (68). Thus, increased adenosine levels following an inflammatory episode could activate the P1 receptors, exerting anti-inflammatory effects through modulation of the NLRP3 inflammasome, thus, contributing to the resolution of gout flares.

Currently, only limited evidence is available to directly demonstrate the involvement of P1 receptors in suppressing gout inflammation or promoting gout resolution. However, based on the anti-inflammatory effect of the P1 receptor signaling pathway, we can see an inevitable impact on the pathogenesis of gout through changes in adenosine concentrations during the different stages of gout flares.

## Alterations in Purine Signaling Pathway During Gout Flares

As previously mentioned, the purine signaling pathway plays a different role in the inflammatory regulation of gout, with ATP playing a central role in its initiation. The type and concentration of purinergic metabolites during a gout flare are altered by various mediators in the inflammatory environment, which can influence the onset and resolution of gout.

### Conversion of Purinergic Metabolites During Gout Flares

MSU alone is not sufficient for initiating a gout flare; the synergistic effect of high levels of extracellular ATP is also required. After the initiation of gout, an increased expression of nucleoside triphosphate dihydrolase 1 (CD39) and 5’-nucleotidase (CD73) can be seen in an inflammatory environment. CD39 dephosphorylates extracellular ATP to ADP and adenosine monophosphate (AMP), which is further converted to adenosine by CD73 (69, 70). Thus, the changes in the expression of CD39 and CD73 during a gout flare can determine the level of nucleotides and the state of inflammation that affects the course of gout disease.

The CD39 and CD73 are extracellular nucleotidases expressed on monocytes, macrophages, B cells, T cells, natural killer cells, dendritic cells, and neutrophils. They mainly maintain a balance between the extracellular ATP and the concentration of adenosine. This homeostasis is important for the persistence and extent of the inflammatory state (71). Changes in the levels of nucleotide enzymes can occur during gout flares. Inflammatory cytokines, oxidative stress, and a hypoxic environment may increase the activity of CD39 and CD73 (72), thereby reducing ATP levels and increasing adenosine concentration. Further, tissue hypoxia can also reduce the expression of nucleoside transporters, increasing the adenosine levels. CD39 is the main enzyme that metabolizes ATP. *In vitro* studies in macrophages have found that activated P2X_7_ receptor could upregulate the expression of cytosolic CD39 by triggering a lipid raft-dependent mechanism (73). In contrast, the elevated CD39 could limit the P2X_7_R-mediated pro-inflammatory response. The knockdown of CD39 results in excessive IL-1β release (74, 75). This implies that in addition to catabolizing ATP and promoting adenosine production, the interaction between CD39 and the P2X_7_ receptor can modulate the upregulated inflammatory state of the organism in time to restore cellular homeostasis.

Macrophages are the primary sites of signaling events that regulate the onset and remission of gout. During the disease progression, macrophages show a shift from a pro-inflammatory M1 phenotype to an anti-inflammatory M2 phenotype. Zanin et al. found (76) that M1 macrophages showed a decrease in the expression of CD39 and CD73 along with a reduction in ATP and AMP hydrolysis, whereas the M2 macrophages displayed higher CD39 and CD73 expression, as well as increased ATP and AMP hydrolysis. Also, the addition of adenosine led to an increase in the expression of the M2 macrophage gene, which in turn promoted the conversion of M1 phenotype to M2 phenotype (77). Moreover, after the initiation of inflammation, adenosine was found to decrease the production of pro-inflammatory cytokines and increase the secretion of anti-inflammatory cytokines by macrophages (78). These data suggested that purinergic signaling may be involved in gout resolution by regulating the conversion of macrophages from the M1 to M2 phenotype.

### Changes of Purinergic Receptors During Gout Flares

Besides changes in the purine metabolites, the expression of purinergic receptors seems to be altered in response to the internal environment during the inflammatory process of gout flares. Among them, the changes in the expression of the P2X_7_ receptor have the most important impact. In addition to directly activating the NLRP3 inflammasome, MSU was found to stimulate the expression of P2X_7_ receptors and P2X_4_ receptors, secreting IL-1β and causing gout flares (11). Besides, an interesting experiment found that ethanol can upregulate the expression of P2X_7_ receptors to induce NLRP3 inflammasome activation (79), which partly explains why alcohol consumption predisposes to gout flares.

The expression of the P2Y receptor is also altered during a gout flare. The MSU stimulation of human keratinocytes resulted in an increased expression of P2Y_6_ receptors and P2Y_14_ receptors (45, 53). The P2Y_6_ receptor belongs to the P2Y_1_-like receptor family, which promotes the inflammatory responses and the upregulation by MSU facilitates the initiation and persistence of gout. In contrast, the P2Y_14_ receptor belonging to the P2Y_12_-like receptors inhibits cAMP and exhibits an inflammatory suppressive effect. The upregulation of P2Y_14_ by MSU appears to be detrimental to the development of gout. However, its upregulation can also promote gout pathogenesis through the negative effect of cAMP on the NLRP3 inflammasome (54).

The transition of macrophages from the phenotypes M1 to M2 promotes remission of gout inflammation. Upon macrophage activation, an increase was observed in the expression of P1 receptors (A_1_ and A_3_), which was accompanied by a decrease in the production of pro-inflammatory cytokines IL-6 and TNF-α and an increase in the production of anti-inflammatory factor IL-10. This promoted macrophage polarization and contributed to the suppression of inflammation (80, 81).

### Modulation of Purinergic Signaling in Different Stages of Gout Flares

**Figure 1:**
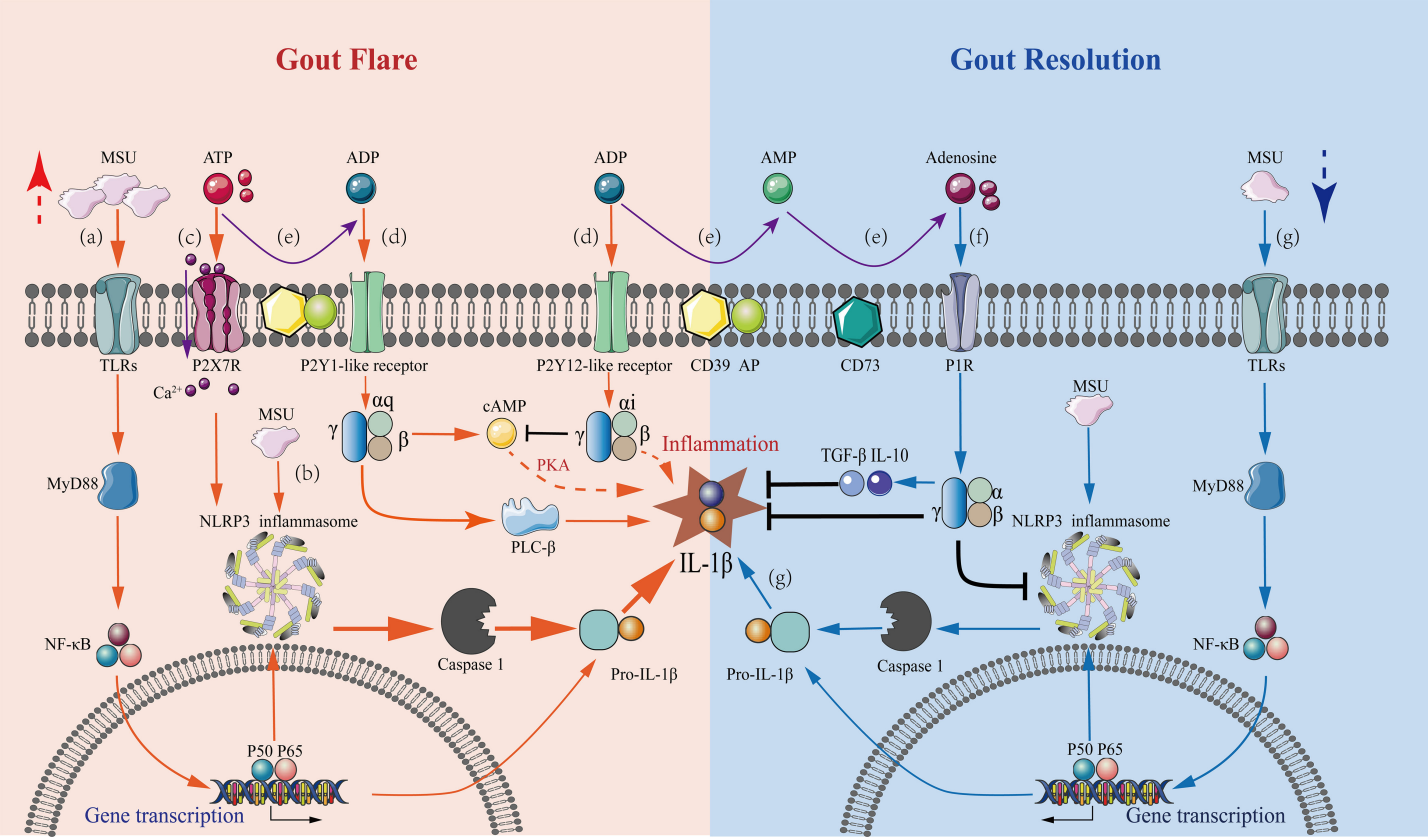
Mechanisms underlying purinergic signaling pathways in the regulation of gout flares and resolution. A series of purinergic receptor-mediated intracellular signaling events in macrophages are involved in gout flare and resolution, with the regulation of IL-1β levels being a central event. Gout flare: **(A)** The binding of extracellular MSU to TLR induces intracellular transcription and accumulation of the pro-IL-1β gene through activation of MyD88-NFκB signaling. **(B)** The uptake of MSU then activates the NLRP3 inflammasome, releasing active caspase-1, which cleaves pro-IL-1β to mature IL-1β. **(C)** Increased extracellular ATP binds to P2X_7_ receptors causing Ca^2+^ influx, which stimulates the NLRP3 inflammasome activation in concert with MSU, leading to massive IL-1β secretion. **(D)** Extracellular ATP and ADP stimulate the P2Y_1_-like receptors coupled to Gq, activating the PLC-IP3/DAG-PKC signaling pathway and cAMP-PKA pathway, which promotes the release of IL-1β. The stimulation of P2Y_12_-like receptors coupled to G_i_ exerts an inhibitory effect on the AC-cAMP-PKA pathway reducing the IL-1β production. However, inhibition of IL-1β production *via* other pathways also exists. Gout resolution: **(E)** In an inflammatory hypoxic environment, the activation of CD39 and CD73 during gout flares results in the progressive degradation of ATP and ADP to adenosine. **(F)** The resultant adenosine activates P1 receptors, decreasing the secretion of pro-inflammatory cytokine IL-1β, thus, promoting the production of anti-inflammatory cytokines TGF-β1 and IL-10. **(G)** Under the inflammatory conditions of gout, ATP is degraded, which affects the sustained stimulation of the P2X_7_R-NLRP3 signaling pathway. This results in a marked reduction of IL-1β secretion by MSU stimulation alone, which is not sufficient to sustain gout, and the condition then tends toward inflammatory remission.

The previous section showed *in vivo* conversions of purine metabolites at different stages of a gout flare. Early in the flare, large amounts of ATP were released extracellularly by the necrotic, apoptotic, and inflammatory cells through pannexins or connexin channels (82). First, ATP was bound to the P2X_7_ receptors in the cytosol. Then, in concert with MSU, it stimulated the activation of the NLRP3 inflammasome, thus, prompting IL-1β secretion. Next, the expressions of CD39 and CD73 were increased in the inflammatory hypoxic environment following gout initiation, which promoted the dephosphorylation of ATP to adenosine. This inhibited the inflammatory response and promotes the self-resolution of gout flares by activating the P1 receptor signaling pathway. During the process, changes in the expression of purinergic receptor induced by the internal environment contributed to the ordered changes in purine signaling that regulated both gout flare and resolution (**Figure 1**). The main reasons for this seemingly contradictory mechanism are the ordered changes in the expression of purine metabolites and their receptors in the inflammatory environment, as well as the different purine signaling pathways they mediate in response to inflammation. Also, the accumulation of acidic metabolites in the inflammatory environment inhibits the cAMP/PKA signaling pathway reducing the IL-1β production (83), which indirectly counteracts the purinergic signaling-mediated cAMP/PKA pathway activity, favoring gout resolution.

Gout is a self-limiting disease with numerous causes contributing to its self-resolution. It is widely recognized that after initiating the inflammatory response in gout, neutrophils phagocytose MSU to form NETs. The aggregated NETs act by reducing the expression of pro-inflammatory cytokines (IL-1β, IL-6, IL-8), which promote the production of anti-inflammatory cytokines (TGF-β1, IL-1Ra, IL-10, IL-37), attenuating the inflammatory response (1, 84). Additionally, CD14 plays a role in the self-relief of gout. The knockdown of CD14 can reduce the activation of MSU-induced NLRP3 and release IL-1β (85). Studies have observed that a reduction of CD14 expression in gout patients may contribute to gout resolution (86). Besides neutrophils and CD14, purinergic signaling pathways play a synergistic role in gout resolution through the regulation of inflammatory responses.

## Conclusion and Outlook

In summary, purinergic signaling pathways are involved in regulating the entire process of gout flare and resolution. After the initiation of the inflammatory response in gout, inflammation-induced microenvironmental changes lead to an orderly alteration in the type of purinergic metabolites in the body, gradually converting a large amount of ATP released from necrotic cells into ADP and adenosine. These purinergic metabolites stimulate the corresponding purinergic receptors and exert different regulatory effects on the inflammatory response. With the conversion of ATP to adenosine, the pro-inflammatory response mediated by the P2X and P2Y receptor signaling pathway shifts to an anti-inflammatory effect mediated mainly by the P1Y receptor signaling pathway, which then balances and restores the body’s homeostasis. These changes may therefore be an important mechanism of action for self-resolution of gout flares.

The purinergic signaling pathways can be potential targets for future interventions in gout pathogenesis. Currently, attempts are made to use targeted purinergic receptor therapy in gout. In a recent study, researchers designed and synthesized a novel P2Y_14_ receptor antagonist, which reduced the MSU-induced joint swelling and inflammatory infiltration in an acute gouty arthritis mouse model (87, 88), thus, improving the clinical value of targeted purinergic signaling in gout. However, the regulation of inflammation by purinergic signaling is much more complex than expected. It is not only manifested by the complexity of purinergic receptor-mediated signaling mechanisms, for example, the same receptor exhibiting opposite effects on inflammation in different settings, but it is also manifested by the diversity of their ligands. Besides activation by purinergic metabolites, receptors can be activated by additional endogenous ligands. A complex regulation network is formed between the endogenous ligands and P2Y receptors, finely tuning the nucleotide receptor signaling pathway (89). Therefore, the internal environment also has an important influence on purinergic signaling. An in-depth study of the regulatory mechanisms underlying different purinergic signals and the integration of various upstream and downstream influencing factors can accurately determine the role of purinergic signaling in gout flares, providing effective strategies for clinical intervention.

## Author Contributions

XL wrote the manuscript. JG drafted the figures. JT contributed to provide the general idea and edited the manuscript. All authors contributed to the article and approved the submitted version.

## Funding

This work was supported by the National Natural Science Foundation of China (81771774) and Anhui Provincial Key research and development plan (201904a07020103).

## Conflict of Interest

The authors declare that the research was conducted in the absence of any commercial or financial relationships that could be construed as a potential conflict of interest.

## Publisher’s Note

All claims expressed in this article are solely those of the authors and do not necessarily represent those of their affiliated organizations, or those of the publisher, the editors and the reviewers. Any product that may be evaluated in this article, or claim that may be made by its manufacturer, is not guaranteed or endorsed by the publisher.
