# Supplementary material for: Purinergic Signaling in the Regulation of Gout Flare and Resolution
Source: Front Immunol. 2021 Dec 1;12:785425. doi: 10.3389/fimmu.2021.785425 (PMC8671294; doi:10.3389/fimmu.2021.785425)
Supplement: Supplementary file 1 [file Table_1.pdf]

Supplementary Table 1. Purinergic receptors

Classification	Typing	Ligands	G-protein coupling	Intracellular signal transduction	Distribution on immune Cells	Agonist or antagonist
P1R	A1	Adenosine / inosine, AMP	Gi	↓AC-cAMP-PKA	Monocytes, macrophages, dendritic cells, neutrophils,	PSB-36 (antagonist) [1]; DPCPX (antagonist) [2]; Neladenoson (agonist) [3]
	A2A	Adenosine / inosine	Gs	↑AC-cAMP-PKA	Monocytes, macrophages, dendritic cells, neutrophils,T cells, B cell	SCH (antagonist) [2]; KW6002 (antagonist) [4]; Regadenoson (agonist) [3]
	A2B	Adenosine	Gs, Gq	↑AC-cAMP-PKA	Monocytes, macrophages, dendritic cells,mast cells,	P453 (agonist) [5]; MRS 1754 (antagonist) [6]
	A3	Adenosine / inosine	Gi	↓AC-cAMP-PKA	Monocytes, macrophages, dendritic cells, neutrophils,	2-Cl-IBMECA (agonist) [6]; MRS1523 (antagonist) [6]; PSB-10 (antagonist) [1]
P2XR	P2X1	ATP	/	Ionic channels open	Monocytes, macrophages, T cells, dendritic cells, neutrophils, B cell	NF449 and NF279 (antagonist)[7]; ATA (antagonist) [8]
	P2X2	ATP	/	Ionic channels open	Mast cells, B cell	NF770 (antagonist) [9]
	P2X3	ATP	/	Ionic channels open	B cell	ATA (antagonist) [8]; A-317491 (antagonist) [10]; MK7264 (antagonist) [11];
	P2X4	ATP	/	Ionic channels open	Monocytes, macrophages, dendritic cells, neutrophils, Mast cells, T cells, B cell	NC-2600 (antagonist) [12]; 5-BDBD (antagonist) [13]
	P2X5	ATP	/	Ionic channels open	Monocytes, macrophages, dendritic cells, neutrophils,T cells, B cell	/
	P2X6	ATP	/	Ionic channels open	Macrophages, B cell	/
	P2X7	ATP	/	Ionic channels open	Monocytes, mast cells, macrophages, microglia, dendritic cells, T cell, B cell	JNJ-54175446 (antagonist) [12]; A740003 (antagonist) [14]
P2YR	P2Y1	ADP, ATP	Gq	↑PLC-IP3/DAG-PKC	Monocytes, dendritic cells, macrophages, T cell, B cell	MRS2365 (agonist) [15]; MRS2179 (antagonist) [10];

P2Y2	UTP, ATP	Gq	↑PLC-IP3/DAG-PKC	Macrophage, neutrophils, epithelial cells, T cell, B cell	DQS (agonist) [16];<br>MRS2698 (agonist) [17];<br>ARC118925XX (antagonist) [16,18];
P2Y4	UTP, ATP	Gq, Gi	↑PLC-IP3/DAG-PKC	Monocyte,macrophages,endothelial cells, T cell, B cell	MRS4062 (agonist) [17];<br>PSB-16133 (antagonist) [17]
P2Y6	UDP, UTP	Gq	↑PLC-IP3/DAG-PKC	Monocytes, macrophage,stromal cells, epithelial cells, T cell, B cell	MRS2578(antagonist) [17]
P2Y11	UTP, ATP	Gq, Gs	↑PLC-IP3/DAG-PKC<br>↑AC-cAMP-PKA	Monocytes, dendritic cells, macrophages,T cell, B cell	NF546 (agonist) [19];<br>NF340 (antagonist) [20];
P2Y12	ADP, ATP	Gi	↓AC-cAMP-PKA	Platelets, monocytes, macrophage, T cell, B cell	Clopidogrel (antagonist) [3];<br>AZD1283 (antagonist) [21]
P2Y13	ADP, ATP	Gi	↓AC-cAMP-PKA	Distinct cell, monocytes, T cell, B cell	MRS2211 (antagonist)[22]
P2Y14	UDP-sugars，UDP，	Gi	↓AC-cAMP-PKA	Epithelial cells, distinct cell, neutrophils,T cell, monocytes, B cell	MRS2905 (agonist) [17];<br>PPTN (antagonist) [17]

Supplementary References

[1] Saze Z, Schuler PJ, Hong CS, Cheng D, Jackson EK, Whiteside TL. Adenosine production by human B cells and B cell-mediated suppression of activated T cells. *Blood*. (2013) 122:9-18. doi: 10.1182/blood-2013-02-482406.
[2] Huang W, Bai S, Zuo X, Tang W, Chen P, Chen X, et al. An adenosine A1R-A2aR imbalance regulates low glucose/hypoxia-induced microglial activation, thereby contributing to oligodendrocyte damage through NF-κB and CREB phosphorylation. *Int J Mol Med*. (2018) 41:3559-69. doi: 10.3892/ijmm.2018.3546.
[3] Wernly B, Zhou Z. More purinergic receptors deserve attention as therapeutic targets for the treatment of cardiovascular disease. *Am J Physiol Heart Circ Physiol*. (2020) 319:H723-29. doi: 10.1152/ajpheart.00417.2020.
[4] Miao Y, Chen X, You F, Jia M, Li T, Tang P, et al. Adenosine A2A receptor modulates microglia-mediated synaptic pruning of the retinogeniculate pathway during postnatal development. *Neuropharmacology*. (2021) 108806. doi: 10.1016/j.neuropharm.2021.108806.
[5] Fusco I, Cherchi F, Catarzi D, Colotta V, Varano F, Pedata F, et al. Functional characterization of a novel adenosine A2B receptor agonist on short-term plasticity and synaptic inhibition during oxygen and glucose deprivation in the rat CA1 hippocampus. *Brain Res Bull*. (2019) 151:174-80. doi: 10.1016/j.brainresbull.2019.05.018.
[6] Ohsawa K, Sanagi T, Nakamura Y, Suzuki E, Inoue K, Kohsaka S. Adenosine A3 receptor is involved in ADP-induced microglial process extension and migration. *J Neurochem*. (2012) 121:217-27. doi: 10.1111/j.1471-4159.2012.07693.x.
[7] Soare AY, Malik HS, Durham ND, Freeman TL, Alvarez R, Patel F, et al. P2X1 Selective Antagonists Block HIV-1 Infection through Inhibition of Envelope Conformation-Dependent Fusion. *J Virol*. (2020) 94:e01622-19. doi: 10.1128/JVI.01622-19.
[8] Obrecht AS, Urban N, Schaefer M, Röse A, Kless A, Meents JE, Lampert A, Abdelrahman A, Müller CE, Schmalzing G, Hausmann R. Identification of aurintricarboxylic acid as a potent allosteric antagonist of P2X1 and P2X3 receptors. *Neuropharmacology*. (2019) 158:107749. doi: 10.1016/j.neuropharm.2019.107749.
[9] Wolf C, Rosefort C, Fallah G, Kassack MU, Hamacher A, Bodnar M, et al. Molecular determinants of potent P2X2 antagonism identified by functional analysis, mutagenesis, and homology docking. *Mol Pharmacol*. (2011) 79:649-61. doi: 10.1124/mol.110.068700.
[10] King BF. P2X3 receptors participate in purinergic inhibition of gastrointestinal smooth muscle. *Auton Neurosci*. (2021) 234:102830. doi: 10.1016/j.autneu.2021.102830.
[11] Richards D, Gever JR, Ford AP, Fountain SJ. Action of MK-7264 (gefapixant) at human P2X3 and P2X2/3 receptors and in vivo efficacy in models of sensitisation. *Br J Pharmacol*. (2019) 176(13):2279-91. doi: 10.1111/bph.14677.
[12] Jacobson KA, IJzerman AP, Müller CE. Medicinal chemistry of P2 and adenosine receptors: Common scaffolds adapted for multiple targets. *Biochem Pharmacol*. (2021) 187:114311. doi: 10.1016/j.bcp.2020.114311.
[13] Han SJ, Lovaszi M, Kim M, D'Agati V, Haskó G, Lee HT. P2X4 receptor exacerbates ischemic AKI and induces renal proximal tubular NLRP3 inflammasome signaling. *FASEB J*. (2020) 34:5465-82. doi: 10.1096/fj.201903287R.
[14] Karasawa A, Kawate T. Structural basis for subtype-specific inhibition of the P2X7 receptor. *Elife*. (2016) 9;5:e22153. doi: 10.7554/eLife.22153.
[15] Bourdon DM, Mahanty SK, Jacobson KA, Boyer JL, Harden TK. (N)-methanocarba-2MeSADP (MRS2365) is a subtype-specific agonist that induces rapid desensitization of the P2Y1 receptor of human platelets. *J Thromb Haemost*. (2006) 4:861-8. doi: 10.1111/j.1538-7836.2006.01866.x.
[16] Endo KI, Sakamoto A, Fujisawa K. Diquafosol tetrasodium elicits total cholesterol release from rabbit meibomian gland cells via P2Y2 purinergic receptor signalling. *Sci Rep*. (2021) 11(1):6989. doi: 10.1038/s41598-021-86433-6.
[17] Jacobson KA, Delicado EG, Gachet C, Kennedy C, von Kügelgen I, Li B, et al. Update of P2Y receptor pharmacology: IUPHAR Review 27. *Br J Pharmacol*. (2020) 177:2413-33. doi: 10.1111/bph.15005.
[18] McEwan TB, Sophocleous RA, Cuthbertson P, Mansfield KJ, Sanderson-Smith ML, Sluyter R. Autocrine regulation of wound healing by ATP release and P2Y2 receptor activation. *Life Sci*. (2021) 283:119850. doi: 10.1016/j.lfs.2021.119850.
[19] Prada MP, Syed AU, Reddy GR, Martín-Aragón Baudel M, Flores-Tamez VA, et al. AKAP5 complex facilitates purinergic modulation of vascular L-type Ca2+ channel CaV1.2. *Nat Commun*. (2020) 11(1):5303. doi: 10.1038/s41467-020-18947-y.
[20] Liu CL, Cheng SP, Chen MJ, Lin CH, Chen SN, Kuo YH, et al. Quinolinate Phosphoribosyltransferase Promotes Invasiveness of Breast Cancer Through Myosin Light Chain Phosphorylation. *Front Endocrinol (Lausanne)*. (2021)

11:621944. doi: 10.3389/fendo.2020.621944.
[21] Kong D, Xue T, Guo B, Cheng J, Liu S, Wei J, et al. Optimization of P2Y12 Antagonist Ethyl 6-(4-((Benzylsulfonyl)carbamoyl)piperidin-1-yl)-5-cyano-2-methylnicotinate (AZD1283) Led to the Discovery of an Oral Antiplatelet Agent with Improved Druglike Properties. *J Med Chem*. (2019) 62:3088-3106. doi: 10.1021/acs.jmedchem.8b01971.
[22] Shen D, Shen X, Schwarz W, Grygorczyk R, Wang L. P2Y13 and P2X7 receptors modulate mechanically induced adenosine triphosphate release from mast cells. *Exp Dermatol*. (2020) 29:499-508. doi: 10.1111/exd.14093.
